# Clinical Validity and Utility of Tumor-Infiltrating Lymphocytes in Routine Clinical Practice for Breast Cancer Patients: Current and Future Directions

**DOI:** 10.3389/fonc.2017.00156

**Published:** 2017-08-03

**Authors:** Lironne Wein, Peter Savas, Stephen J. Luen, Balaji Virassamy, Roberto Salgado, Sherene Loi

**Affiliations:** ^1^Peter MacCallum Cancer Centre, University of Melbourne, Melbourne, VIC, Australia; ^2^Department of Pathology, GZA Ziekenhuizen, Antwerp, Belgium; ^3^University of Melbourne, Melbourne, VIC, Australia

**Keywords:** tumor-infiltrating lymphocytes, breast cancer, cancer biomarkers, antitumor immunity, checkpoint inhibitors

## Abstract

The interest in tumor-infiltrating lymphocytes (TILs) as a prognostic biomarker in breast cancer has grown in recent years. Biomarkers must undergo comprehensive evaluation in terms of analytical validity, clinical validity and clinical utility before they can be accepted as part of clinical practice. The International Immuno-Oncology Biomarker Working Group has developed a practice guideline on scoring TILs in breast cancer in order to standardize TIL assessment. The prognostic value of TILs as a biomarker in early-stage breast cancer has been established by assessing tumor samples in thousands of patients from large prospective clinical trials of adjuvant therapy. There is a strong linear relationship between increase in TILs and improved disease-free survival for triple-negative and HER2-positive disease. Higher levels of TILs have also been associated with increased rates of pathological complete response to neoadjuvant therapy. TILs have potential clinical utility in breast cancer in a number of areas. These include prediction of responders to immune checkpoint blockade, identification of primary HER2-positive and triple-negative patients who have excellent prognoses and may thus be appropriate for treatment de-escalation, and potentially incorporation into a neoadjuvant endpoint which may be a better surrogate maker for drug development.

## Introduction

It has been well established that the immune system plays a crucial role in host defense against tumor progression. Unlike solid tumor types such as melanoma, breast cancer is not usually considered an immunogenic; however, recent evidence suggests that tumor immunogenicity is important in the biology of breast cancer and its response to treatment. Tumor-infiltrating lymphocytes (TILs) have provided insight into the immunogenicity of breast tumors and have been shown to be useful as a prognostic biomarker in early-stage triple-negative and HER2-positive breast cancer.

## Background: The Immune System and TILs in Breast Cancer

Cancer immunoediting is believed to be a central component in the evolutionary processes that shape an established tumor, and therefore also plays a critical role in the determination of the resulting immunogenic phenotype (1, 2). Immunoediting comprises three processes, namely, elimination, equilibrium, and escape. Elimination encompasses the concept of immunosurveillance, where the host immune system recognizes tumor antigens and destroys the developing tumor. It is characterized by the presence of CD8+ T lymphocytes, CD4+ type 1 helper T lymphocytes, and natural killer cells. When this elimination process is not successful, the tumor enters the equilibrium phase, whereby the tumor cell clones with superior survival abilities are selected in a Darwinian fashion. Escape occurs when the immunologically sculpted tumor proliferates in an uncontrolled manner and becomes clinically detectable. During escape, FOXP3+ T regulatory lymphocytes, CD4+ type 2 T helper lymphocytes, and myeloid-derived suppressor cells can be seen in the tumor microenvironment (3).

Tumor-infiltrating lymphocytes are immune cells that have been observed in many solid tumors, including breast cancer. They are a population of cells comprising a mixture of cytotoxic T cells and helper T cells, as well as B cells, macrophages, natural killer cells, and dendritic cells. Whilst not discussed extensively in this manuscript, gene expression and flow cytometry profiles demonstrate that TIL quantity, as assessed semiquantitatively on a hematoxylin and eosin (H&E) stained slide, represents a surrogate for a pre-existing favourable host anti-tumor activated T cell response (4, 5).

Tumor-infiltrating lymphocytes are more commonly observed at higher levels in triple-negative and HER2-positive tumors compared with estrogen receptor positive, HER2-negative breast cancer (6–8). This indicates that TNBC and HER2-positive subtypes are more immunogenic, and for reasons yet to be fully elucidated are able to generate more robust T cell anticancer responses. Lately the clinical importance of the presence of TILs has been reported, with associations with improved prognosis and higher response rates to neoadjuvant therapy in the early-stage setting (9, 10).

## What Makes a Good Biomarker?

Biomarkers in cancer must undergo thorough evaluation in terms of analytical validity, clinical validity, and clinical utility before they can be adopted as part of clinical practice (11). Analytical validity refers to the robustness of the test, in terms of accuracy, reliability, and interobserver reproducibility (12). The clinical validity relates to the extent to which the biomarker is associated with prognosis or response to treatment and refers to the ability of a tumor biomarker to divide one population into groups that differ either biologically or clinically. Importantly, clinical validity alone is insufficient to recommend that the biomarker be used to guide clinical treatment decisions. Clinical utility is the usefulness of the biomarker in terms of directing clinical decisions and patient management.

## Analytical Validity

As the interest in TILs as an immunological biomarker grows, so too does the need for standardizing TIL evaluation. The International Immuno-Oncology Biomarker Working Group has developed a practice guideline and tutorial on how to score TILs in breast cancer (13). Currently, most clinical research groups world wide interested in TIL evaluation in breast cancer are using this guideline in their studies, which will promote comparability of studies. This will avoid a plethora of different methods assessing the same biomarker, as has been the experience with Ki-67, although for Ki-67 international efforts at standardization are also underway (14).

Tumor-infiltrating lymphocytes in breast cancer lesions can be seen in both the intratumoral and stromal areas. Intratumoral TILs have cell-to-cell contact with carcinoma cells, while stromal TILs are located in the fibrous stroma adjacent to the tumor cells. Evaluation of intratumoral TILs is generally more difficult and less reproducible than evaluation of stromal TILs but the separation of these two variables may have biological connotations. The current recommendation is therefore to use the percentage of stromal TILs as the principal parameter, where the numerator is the area occupied by TILs and the denominator is the total stromal area, not the number of stromal cells. All mononuclear cells (lymphocytes and plasma cells) are scored, but polymorphonuclear white blood cells are excluded (13).

**Figure 1:**
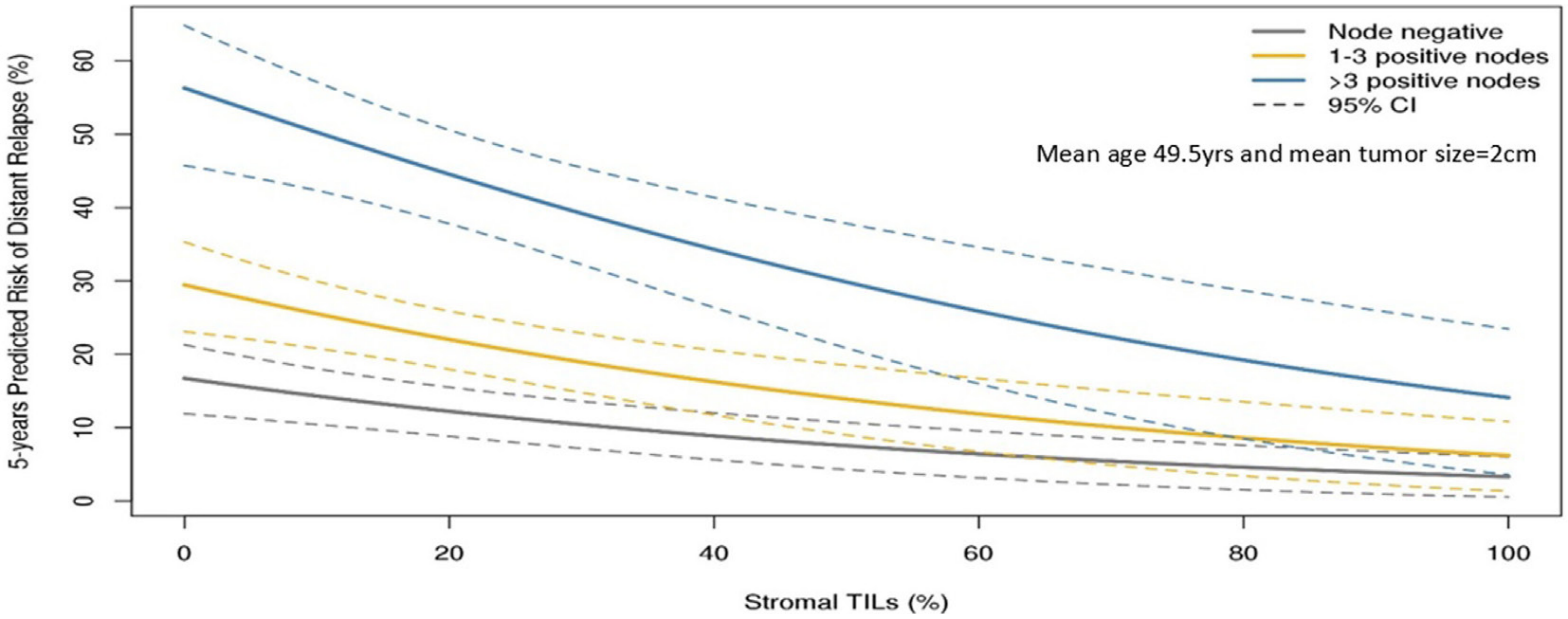
Model to predict risk of distant recurrence at 5 years (%) by tumor-infiltrating lymphocytes (TILs) and nodal status by tumor size and age. The predicted rate of distant recurrence on the *y* axis as a continuous function of the level of stromal TILs in the primary tumor on the *x* axis is presented according to nodal status after adjuvant chemotherapy. This prognostic algorithm is for TNBC and assumes a mean age of 49.5 years and mean tumor size of 2 cm. Loi et al., San Antonio Breast Cancer Symposium 2015 (15).

The guidelines of the International Immuno-Oncology Working Group clearly state that TILs should be assessed as a continuous variable rather than as a categorical variable (13). Having TILs documented as a continuous variable enables it to be implemented in algorithms encompassing other prognostic clinical–pathological variables. Loi and colleagues have demonstrated that TILs incorporated as such in an algorithm containing nodal status, tumor size, and age enables making predictions of the 5-year predicted distant disease-free survival (15) (Figure 1). Where a binary variable is needed, the term lymphocyte predominant breast cancer (LPBC) can be used to describe cancers which comprise more lymphocytes than tumor cells. Previous reports of LPBC have variably utilized a threshold between 50 and 60% stromal lymphocytes; however data since the original publications suggest that this may not be the best cutpoint as this level of immune infiltration represents less than 10% of primary breast cancers. This is considerably lower than that of melanoma and non-small cell lung cancer in which this level of lymphocytic infiltration is present in around 25% of tumors.

Tumor-infiltrating lymphocytes can be easily and are most commonly assessed using light microscopy of H&E stained slides. More complex techniques have been used, such as flow cytometry (16) and multicolor immunohistochemical staining with multispectral imaging (17), as well as gene expression analysis measured using quantitative polymerase chain reactions, gene expression microarrays, and RNA sequencing (18, 19). These techniques can provide more information regarding the different lymphocyte subpopulations; however, gene expression data are limited with regard to identifying specific T cell subsets. All of these techniques remain in the research setting due to cost, lack of robust analytical validity, and requirements for certain types of tissue processing.

The International Immuno-Oncology Working Group conducted two ring studies to evaluate interobserver variability of TIL assessment (20). The aim was to calculate the interclass correlation coefficient (ICC) for assessment of TILs by different pathologists. In the first ring study, 34 pathologists assessed 60 slides for stromal TILs. While the interobserver agreement was relatively good, the prespecified endpoint of an ICC above 0.7 was not reached (ICC: 0.70; 95% CI: 0.62–0.78). A software-guided image evaluation system was developed for the second ring study, where 28 pathologists evaluated 60 slides. The ICC improved to 0.89 (95% CI: 0.85–0.92). This suggests that TIL evaluation can be reproducible among pathologists and that computer-assisted systems may be valuable in this regard. A study by Swisher et al. (21) also found that TIL evaluation can be undertaken with acceptable interobserver agreement. Four pathologists assessed 75 TNBC specimens. The kappa statistic for interobserver agreement was 0.57, which was interpreted as moderate agreement.

**Figure 2:**
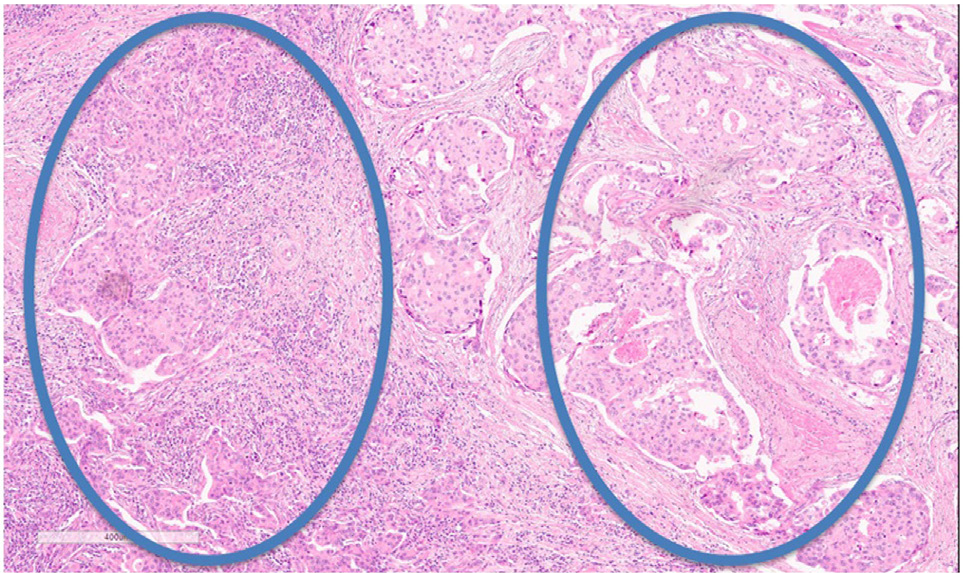
Tumor-infiltrating lymphocyte (TIL) heterogeneity. Zone with high TILs on the left, zone with low TILs on the right. Magnification 100×.

Nevertheless, some hurdles still remain with regard to standardizing TIL assessment. For example, TIL infiltration patterns within a single tumor can be quite heterogeneous (Figure 2). It is well known that the eye of the pathologist may not be able to quantify reliably the spatial variation in infiltration patterns. In this respect, machine learning algorithms may potentially be of added value (22). Regardless, TIL evaluation by H&E remains the most pragmatic for many community laboratories in the absence of data supporting other methods.

**Figure 3:**
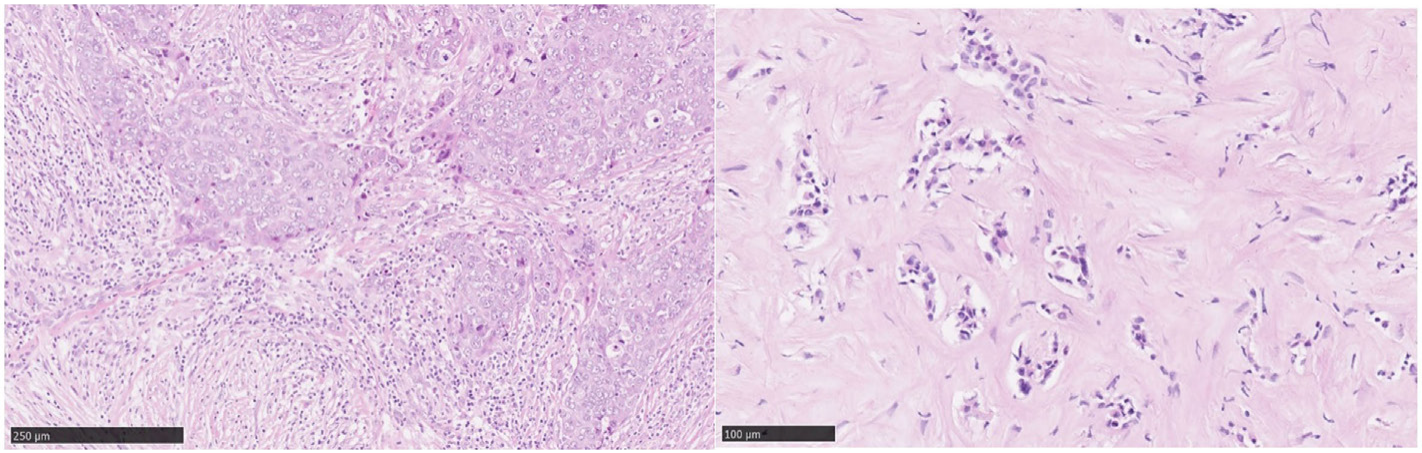
Tumor-infiltrating lymphocytes (TILs): primary versus metastatic disease. Case study: breast primary with high number of TILs (left), pleural metastasis with no TILs (right). Magnification 100× (left), 200× (right).

As the majority of experience in TIL assessment in breast cancer has been in primary lesions, less is known about immune influences in the advanced metastatic setting. Important differences in the immune infiltrates between primary tumors and metastases have recently been reported. Studies of paired primary and metastatic lesions have noted significantly lower levels of TILs in metastases (23–25) (Figure 3). This is in keeping with lower TIL levels described in metastases in lung cancer (26) and renal cell carcinoma (27). The TILs working group guideline (13) methodology for TIL assessment has been used to evaluate TILs in the advanced setting in a retrospective analysis of the CLEOPATRA study (28). This study also found lower TIL levels in metastatic lesions than in their paired primaries, although there were not enough paired samples to adequately power this comparison. The site of metastases may also have an important influence on the immune infiltrate, with higher TIL levels having been observed in lung metastases than in bone, liver, or skin lesions. The International Immuno-Oncology Working Group will soon publish guidelines on scoring TILs in the metastatic setting and in the residual disease setting. Overall, these observations lend evidence to the hypothesis that breast cancer must adapt and learn how to evade the immune system as well as cause significant immunosuppression in the host in order to progress to the advanced disease setting. This has implications for the development of immunotherapies in advanced breast cancer.

## Clinical Validity

### The Prognostic Value of TILs in Early-Stage Breast Cancer

The prognostic value of the TILs biomarker in early-stage breast cancer has been established by assessing tumor samples in patients from large clinical trials (Table 1). There has been shown to be a strong linear relationship between increase in TILs and improved recurrence-free survival for triple-negative breast cancer and HER2-positive breast cancer (10). In the first TIL analysis of node-positive primary breast cancers from the BIG 02-98 trial (7), over 2,000 breast cancer samples were examined for TILs. In triple-negative breast cancer, each 10% increase in intratumoral and stromal TILs was associated with a 17 and 15% reduced risk of relapse, respectively, and 27 and 17% reduced risk of death, respectively, regardless of chemotherapy type.

**Table 1:** Adjuvant trials that have assessed tumor-infiltrating lymphocytes (TILs).

Trial	Treatments	Subtype	*n*	Recurrence end points
BIG 2-98 (7)	Doxorubicin Cyclophosphamide CMF Docetaxel	ER + /HER2− HER2 + TNBC	1,079 297 256	Not significant Not significant For each 10% increment of sTILs: DFS, HR = 0.84 (95% CI: 0.74–0.98, *P* = 0.025)
FinHER (29)	Docetaxel Vinorelbine FEC ±Trastuzumab	ER + /HER2− HER2 + TNBC	591 209 134	Not significant Predictive for higher trastuzumab benefit (*P* interaction = 0.025) For each 10% increment of sTILs: DDFS, HR = 0.79 (95% CI: 0.64–0.98, *P* = 0.032)
E2197 and E1199 (31)	Doxorubicin Cyclophosphamide Docetaxel	TNBC	481	For each 10% increment of sTILs: DFS, HR = 0.84 (95% CI 0.74–0.95, *P* = 0.005)
IBCSG 22-00 (32)	CMF Anthracycline Taxanes CM-maintenance	TNBC	647	For each 10% increment of sTILs: Breast cancer-free interval, HR = 0.87 (95% CI 0.79–0.95, *P* = 0.003)
Pooled analysis (33)	Anthracycline Taxane	TNBC	991	Studies included ECOG2197, ECOG1199, BIG2-98, FinHER, Gustave Roussy For each 10% increment of sTILs: IDFS HR = 0.86 (95% CI: 0.80–0.93, *P* < 0.0001)
Total patients			4,685	

*DDFS, distant disease-free survival; IDFS, invasive disease-free survival; DFS, disease-free survival; iT-CD8, intratumoral CD8; S-CD8, stromal CD8; sTIL, stromal TIL*.

These findings were validated in an analysis of 935 early-stage breast cancer samples from the FinHER trial (29). In triple-negative breast cancer and in HER2-positive patients randomized to trastuzumab, each 10% increase in TILs was associated with decreased distant recurrence. In the HER2-positive group, higher TILs were also associated with greater magnitude of benefit from the addition of adjuvant trastuzumab. A study looking at gene expression profile of TILs and benefit of trastuzumab in the NSABP B-31 HER2-positive breast cancer clinical trial also found that patients with high expression of TILs genes derived significantly more benefit more from trastuzumab than patients with low-TIL expression (30).

In a study of 506 triple-negative tumors from the adjuvant ECOG trials E2197 and E1199, stromal TILs, as a continuous variable, were associated with improved prognosis (31). Similarly, in a recent analysis of 647 TNBC samples, TILs were associated with an improved prognosis at a median follow-up of 6.9 years (32). For every 10% increase in TILs, the breast cancer-free interval risk reduction was 13% (HR 0.87, 95% CI: 0.79–0.95, *P* = 0.003). Higher TIL levels were also associated with better disease-free survival and overall survival, with risk reductions of 11% (*P* = 0.005) and 17% (*P* < 0.001), respectively.

Loi et al. conducted a pooled analysis with individual patient data from 991 TNBC patients from 6 randomized clinical trials (33). Each 10% increase in stromal TILs was associated with a 14% relative reduction in invasive disease-free survival (HR = 0.86, 95% CI: 0.80–0.93, *P* < 0.0001) Figure 1 and a 17% relative reduction in deaths (HR = 0.83, 95% CI: 0.76–0.91, *P* = 0.0001). TILs therefore represent the first biological prognostic factor for primary TNBC that has been shown to be reproducible and also add robust significant addictive prognostic value to current clinicopathological factors. Consideration into inclusion into staging systems such as AJCC (34) should be considered in the future.

Ali et al. (8) analyzed samples from four studies including more than 12,000 patients. They found that in ER-negative tumors (ER-negative/HER2-positive and triple-negative), the presence of stromal and intratumoral CD8+ lymphocytes was associated with improved breast cancer specific survival. However, in ER-positive breast cancer, CD8+ lymphocytes were not found to be associated with breast cancer-specific survival.

The studies discussed above provide robust and consistent evidence that TILs can be used as a prognostic marker in early-stage triple-negative breast cancer and in some patients with HER2-positive disease. At present, the significance of TILs in ER-positive HER2-negative tumors has not been established.

### TILs and Response to Neoadjuvant Chemotherapy

Tumor-infiltrating lymphocytes have been associated with higher rates of pathological complete response (pCR) to neoadjuvant therapy (Table 2). Denkert et al. analyzed 1,058 pretreatment biopsies from two neoadjuvant anthracycline and taxane-based trials and found that the level of intratumoral lymphoctytes was significantly associated with pCR rates (35). In the GeparDuo cohort, the overall pCR rate was 12.8%, while in patients with increased intratumoral lymphocytes (>10%) it was 31%. In the subgroup of LPBC, defined as >60% of either stromal or intratumoral lymphocytes, pCR rate was 41.7%. Similarly, in the GeparTrio cohort, the odds ratio for pCR increased with the extent of TILs. Tumors without any TILS had a pCR rate of 7.2%, while those with LPBC had a pCR rate of 40%.

**Table 2:** Neoadjuvant trials that have assessed tumor-infiltrating lymphocytes (TILs).

Trial	Treatments	Subtype	*n*	pCR
GeparDuo (35)	Doxorubicin Docetaxel Cyclophosphamide	All	218	OR 1.38 of pCR per 10% iTILs (95% CI: 1.08–1.78, *P* = 0.012)
GeparTrio (35)	Doxorubicin Docetaxel Cyclophosphamide Vinorelbine Capecitabine	All	840	OR 1.21 of pCR per 10% iTILs (95% CI: 1.08–1.35, *P* = 0.001)
NeoALTTO (38)	Trastuzumab Lapatinib Paclitaxel FEC	HER2+	387	Every 1% increase in TILs was associated with a 3% decrease in the rate of an event [HR 0.97 (95% CI: 0.95–0.99; *P* = 0.002)] pCR: TILs > 5% associated with higher pCR rates [OR 2.60 (95% CI: 1.26–5.39; *P* = 0.01)]
GeparQuattro (36)	Epirubicin Cyclophosphamide Docetaxel Capecitabine Trastuzumab	HER2 +	156	OR 1.16 of pCR per 10% sTILs (95% CI: 1.01–1.32, *P* = 0.038)
CHER-LOB (37)	Trastuzumab and/or lapatinib Paclitaxel FEC	HER2+	105	Each 10% increase in iTIL and sTIL associated with a higher probability of a pCR (adjusted OR: 2.64, 95% CI: 1.46–4.79, *P* = 0.001 and 1.32 95% CI: 1.08–1.6, *P* = 0.006, respectively)
GeparSixto (19)	Paclitaxel Liposomal doxorubicin Carboplatin Bevacizumab Trastuzumab	HER2+ and TNBC	580	OR 1.2 of pCR per 10% sTILs (95% CI: 1.11–1.29, *P* < 0.001) Significant test for interaction between increased TILs and response to carboplatin therapy
GeparQuinto (39)	Epirubicin Cyclophosphamide Taxane Everolimus	ER+ and TNBC	313	OR 1.2 of pCR per 10% sTILs (95% CI: 1.0–1.3, *P* = 0.01)
EORTC 10994 and BIG 00–01 (40)	FEC Docetaxel	ER-	111	High gTILs: pCR 74.2% Low gTILs: pCR 31.3% OR 6.42 of pCR for high versus low gTILs (95% CI: 2.08–19.83, *P* = 0.001)
Total patients			2,710	

*pCR, pathological complete response; iTIL, intratumoral TIL; sTIL, stromal TIL; gTIL, gene expression surrogate TIL*.

Tumor-infiltrating lymphocytes have been associated with achieving response to neoadjuvant HER2-targeted therapies in several studies. In an analysis of the GeparQuattro trial, each 10% increment in TILs was found to be associated with higher responses to neoadjuvant trastuzumab and chemotherapy (36). Similarly, in a study of 105 HER2-positive tumors from the CHER-LOB trial (37), both intratumoral and stromal TILs were associated with a higher pCR rates (OR: 2.64, 95% CI: 1.46–4.79, *P* = 0.001 and 1.32, 95% CI: 1.08–1.6, *P* = 0.006, respectively).

Additionally, in the NeoALLTO study (38) of HER2-positive patients treated with neoadjuvant HER2-targeted therapies and chemotherapy, TIL levels greater than 5% were associated with higher rates of pCR. This study also found the presence of TILs at diagnosis to be a positive, independent prognostic marker. There was a linear relationship between TILs and event-free survival, with every 1% increase in TILs being associated with a 3% decrease in the rate of an event.

The GeparSixto trial investigated the effect of adding carboplatin to an anthracycline-plus-taxane combination in triple-negative and HER2-positive tumors. Evaluation of these tumors demonstrated that increased levels of stromal TILs and LPBC were associated with higher rates of pCR (19). pCR rates in LPBC tumors were significantly higher with the addition of carboplatin, with carboplatin increasing the odds of pCR 3.71-fold in LPBC tumors compared with a 1.01-fold increase in non-LPBCs.

Lymphocytic infiltrate has also been shown to be associated with response to chemotherapy in HER2-negative disease. In an analysis of 313 biopsies from a substudy of the neoadjuvant GeparQuinto trial, LBPC had a significantly higher pCR rate compared to non-LPBC (36.6 versus 14.3%) (39). In an exploratory analysis, pCR rates were analyzed according to hormone receptor status. In hormone receptor positive tumors, the pCR rate for LPBC was 28.2%, while it was only 8.2% for non-LPBC tumors. Use of TILs as a predictive marker in the setting of hormone receptor-positive HER2-negative disease requires further validation.

West et al. examined 111 ER-negative tumors from patients treated with neoadjuvant anthracycline-based regimens (40). Pathologic complete responses were observed in 74% of TIL-high patients and 31% of TIL-low patients (odds ratio 6.33; 95% CI: 2.49–16.08; *P* < 0.0001).

The association of different subtypes of TILs with achieving response to neoadjuvant chemotherapy has been investigated in TNBC (41). It was found that a high CD8+/FOXP3+ ratio was a strong predictor of pCR. This result is consistent with the immunoediting hypothesis, as this TIL composition is representative of the elimination process.

Note that while a binary LPBC value was used commonly in these studies, present recommendations currently do not propose a specified cutoff for neoadjuvant patients. These findings do suggest that patients with low TILs on their baseline core-biopsy may need to be observed very carefully during their neoadjuvant therapy or perhaps baseline TILs levels could facilitate a decision to go straight to surgery (where possible).

### TILs in Residual Disease

Tumor-infiltrating lymphocytes in residual disease after neoadjuvant therapy have been shown to be associated with a more favorable prognosis (Table 3). In an analysis of 278 TNBC specimens post neoadjuvant treatment, Dieci et al. found the 5-year overall survival for high-TIL patients was 91%, compared to 55% for low-TIL patients (42). Similarly, in a cohort of 111 TNBC patients with residual disease following neoadjuvant chemotherapy, a strong positive linear association was observed between TILs in the residual disease specimen and both recurrence-free survival and overall survival (43).

**Table 3:** Tumor-infiltrating lymphocytes (TILs) in residual disease post neoadjuvant treatment.

Trial	Treatments	Subtype	*n*	End points
Miyashita et al. (41)	Anthracycline Taxane	TNBC	131	CD8(+) TIL had strong prognostic significance for RFS: HR 3.09 (95% CI: 1.537–6.614, *P* = 0.0013) CD8/FOXP3 ratio significantly correlated with RFS: HR 2.07 (95% CI: 1.029–4.436, *P* = 0.0412)
Dieci et al. (42)	Anthracycline Taxane Other	TNBC	278	For each 10% increase in sTIL MFS HR 0.79 (95% CI: 0.71–0.88) and OS HR 0.79 (95% CI: 0.71–0.89), *P* < 0.001 for both outcomes 5-year OS 91% (95% CI: 68–97%) for high-TIL patients, 55% (95% CI: 48–61%) for Low-TIL patients (HR 0.19, 95% CI: 0.06–0.61, *P* = 0.0017)
Loi et al. (43)	Doxorubicin Cyclophosphamide Paclitaxel	TNBC	111	A strong positive linear association of TILs in NAC-treated specimens was observed with RFS (*P* = 0.0001, relative risk reduction of 3.4% for each 1% of TILs) and OS (*P* = 0.0016; relative risk reduction of 2.8% for each% of TILs)
Total patients			520	

*DFS, disease-free survival; MFS, metastases-free survival, NAC, neoadjuvant chemotherapy; OS, overall survival; RFS, recurrence free survival; sTIL, stromal TIL; TNBC, triple-negative breast cancer*.

### The Prognostic Value of TILs in Metastatic Breast Cancer

Tumor immunogenicity is hypothesized to decrease in the advanced setting (Table 4). This is based on the theory of immunoediting (1), and observations that metastatic tumors have lower levels of TILs than their matched primary tumors (24, 44). The prognostic value of TILs in advanced HER2-positive breast cancer has recently been examined (28). Six hundred and seventy-eight tumor samples from the CLEOPATRA study were assessed for TILs. Fifty eight of these were from metastatic sites. Twenty patients had paired primary and metastatic samples. There was no significant association between TIL levels and progression-free survival; however, for overall survival, each 10% increase in TILs was significantly associated with prolonged overall survival. This finding expands the prognostic role of TILs from early-stage disease to the metastatic setting. An additional intriguing finding from this study is that TIL levels vary depending on the ethnicity of the patients implying that host factors may also be important determinants of antitumor immunity. This raises an additional possibility that stratification according to ethnicity, or at least documentation of ethnicity in clinical trials is needed to be able to tease out any effect of ethnicity on response to immuno-therapeutics.

**Table 4:** Tumor-infiltrating lymphocytes (TILs) in metastatic disease.

Trial	Treatments	Subtype	*n*	Survival end points
CLEOPATRA (28)	Docetaxel Trastuzumab ±Pertuzumab	HER2+First line	678 (58 from metastatic sites)	For each 10% increase in sTILs OS adjusted HR 0.89, 95% CI: 0.83−0.96, *P* = 0.0014 No significant association between TILs PFS (adjusted HR 0.95, 95% CI: 0.90−1.00, *P* = 0.063) Median TILs 10%
Kashiwagi et al. (45)	Eribulin	All	52 (all primary lesions)	No significant difference in DFS between high and low-TIL groups (*P* = 0.489) TNBC: high TILs (>10%) group had significantly longer DFS than the low-TIL group (*P* = 0.033)
Schmid et al. (48)	Atezolizumab	TNBC	115	TILs^a^ ≤ 10%: median OS 6.6 months TILs > 10%: median OS 12.6 months
Total patients			845	

*DFS, disease-free survival; OS, overall survival; sTIL, stromal TIL, TNBC, triple-negative breast cancer*.

*^a^TILs were evaluated in this study as immune infiltrate as a percentage of tumor area and not as in the current recommendations*.

The use of TILs in predicting response to eribulin in metastatic breast cancer has recently been evaluated (45). TILs were assessed in primary lesions at the time of breast cancer diagnosis in 52 patients. For TNBC, patients in the high-TIL group had significantly longer disease-free survival than TNBC patients in the low-TIL group. While limited by small numbers, the results of this study indicate that TILs may have a role in predicting response to eribulin in advanced TNBC.

These findings are consistent with the prognostic value of TILs that has been reported in the metastatic setting in other tumor types. Lymphocytic infiltrates in metastatic lesions have been found to be a prognostic marker in metastatic melanoma (46) and colorectal cancer (47). TILs in metastatic breast cancer, while are overall present at lower levels than primary disease, still maintain prognostic value. The advanced setting will be more complex than primary disease with immune heterogeneity likely existing at the different metastatic sites (28).

## Clinical Utility

Tumor-infiltrating lymphocytes have potential clinical utility in breast cancer in a number of different domains. Since TILs are read on simple H&E stained slides, it is not surprising that there is a tremendous interest to investigate their importance for the clinical setting.

We see the clinical utility of TILs in the following ways:

1. Prediction of responders to checkpoint blockade monotherapy.

Schmid et al. (48) recently reported the results of a phase 1a study of atezolizumab monotherapy in 115 metastatic TNBC patients. Higher overall response rates and longer overall survival were observed in patients with higher TIL levels. In the group with TILs ≤ 10%, median OS was 6.6 months, compared with 12.6 months for those with TILs > 10%. Of note, TILs here were evaluated differently and not as in the current guidelines. TILs were defined as immune infiltrate as a percentage of the tumor area. While further refinement of immune markers is likely, TILs evaluated on H&E slides could represent an easy first step for many laboratories to determine which patients may benefit from checkpoint blockade as a single agent. Data in this area will undoubtedly increase in the next few years.

2. Identification of primary HER2+ and TNBC patients with excellent prognoses.

Tumor-infiltrating lymphocytes as a robust prognostic factor may potentially lead to the identification of those patients with excellent prognosis and thus may guide adjuvant treatment de-escalation efforts. For example, in HER2+ patients with high-TIL levels, it may be appropriate to consider using only a taxane and trastuzumab rather than maximal therapy with combination anthracycline, taxane, trastuzumab, and pertuzumab (10). In the pooled analysis of 991 primary TNBC patients, those patients with node negative disease and TILs 20% or more (which is around the average level) had excellent 5-year distant metastases-free survival (92% CI: 87–97%) (Figure 1). TILs may have potential utility in the design of clinical trials, by appropriately grouping patients into those who may benefit from standard treatments, and those who require novel therapeutic strategies (49).

3. Refinement of the neoadjuvant pCR endpoint.

The neoadjuvant model represents an attractive model for drug development (50). What remains to be clearly elucidated is how the change in pCR induced by new agents will correlate with improvements in disease-free survival in the adjuvant setting (51, 52). Given that TILS are prognostic in the residual disease setting, we hypothesize that incorporation of TILS into the pCR/residual disease endpoint may improve prediction of those who do well, and help us understand how a novel agent performs. Incorporation into existing models such as residual cancer burden (53) may improve these surrogate endpoints as not all patients who do not achieve pCR relapse and identification of these patients that most benefit from the new agent will aid adjuvant trial design and statistical calculations.

## Can We Create a TIL Response were None Exists?

A lack of the presence of antitumor immunity bodes for a poor prognosis and poor response to therapy. Therefore, a key area of interest is how to modulate the tumor microenvironment in order to convert a “low-TIL” tumor to a “high-TIL” tumor. Therapeutics could stimulate antigenicity and allow the augmentation of the antitumor immune response with checkpoint inhibitors. Potential approaches include multimodal combinations with chemotherapy, targeted therapies, radiotherapy, personalized vaccines, and adoptive T cell therapies (49).

The combination of conventional chemotherapeutic agents and immunotherapies is currently being investigated. A phase III trial of nab-paclitaxel combined with the anti-PD-L1 inhibitor atezolizumab in advanced TNBC is underway (ClinicalTrials.gov.NCT02425891), as is a phase III study of pembrolizumab plus chemotherapy (ClinicalTrials.gov NCT NCT02819518).

Ras–MAPK pathway activation has been described as a mechanism of promoting immune evasion in TNBC (43). Genetic alterations in Ras–MAPK signaling have been significantly associated with lower TIL levels. MEK inhibition can upregulate major histocompatibility complex and PD-L1 expression in TNBC cells both *in vivo* and *in vitro*. Thus, this combination is a very attractive hypothesis to investigate in patients. The combination of the MEK inhibitor cobimetinib, taxane chemotherapy, and atezolizumab is currently being investigated in advanced TNBC patients (ClinicalTrials.gov NCT02322814).

The efficacy of trastuzumab in HER2-positive breast cancer has traditionally been thought to be due to reductions in signaling downstream of the HER2 receptor. However, it is now understood that antibody-dependent cell-mediated cytotoxicity as well as T cell-mediated cytotoxicity play a central role in its action (54–59). Studies of immunotherapies combined with anti-HER2 treatments are currently recruiting and the results are eagerly awaited (ClinicalTrials.gov NCT02318901, NCT02605915, NCT02924883).

## Conclusion

Tumor-infiltrating lymphocytes, as evaluated on H&E slides, has been shown in numerous studies now to be a reliable and reproducible marker of pre-existing antitumor immunity in breast cancer. It is clear that higher levels of TILs are associated with improved prognosis in early and advanced stage TNBC and HER2-positive breast cancer, as well as a higher probability of achieving pCR in the neoadjuvant setting. Analysis of TILs in residual disease specimens after neoadjuvant therapy has also been shown to have prognostic value. The evaluation of TILs as a biomarker in breast cancer is likely to be extended from the research domain to the clinical setting in the future.

As the body of evidence of the clinical validity of TILs grows, so too does the appreciation of the importance of the immune system in the biology and outcome of breast cancers. There remain considerable challenges in optimizing host antitumor immunity, and combination of checkpoint inhibitors with traditional and novel therapies is an area of active investigation.

## Author Contributions

Preparation/drafting of manuscript: LW, PS, SJL, BV, RS, and SL; figures: LW, RS, and SL.

## Conflict of Interest Statement

The authors declare that the research was conducted in the absence of any commercial or financial relationships that could be construed as a potential conflict of interest.
